# Ceramide Synthase HYL-2 is Required for Neural Preconditioning to Anoxia in *Caenorhabditis elegans* .

**DOI:** 10.17912/micropub.biology.001024

**Published:** 2024-05-29

**Authors:** Ginger Watzinger, Heather L Bennett

**Affiliations:** 1 Department of Biology, Trinity College, Hartford, Connecticut, United States

## Abstract

Oxygen is vital for neuron development and function, and low oxygen (hypoxia) or 0% oxygen available (anoxia) conditions lead to neuronal dysfunction and death. Nonlethal forms of stress, prior to hypoxic or anoxic (preconditioning) environments protects neurons and increases survival to oxygen deprivation. Hyperpolarization of *C. elegans* neurons prior to anoxia (neural preconditioning) increases survival, but the cellular and molecular pathways that confer survival are unclear. Here we report that loss in ceramide synthase gene, *hyl-2* suppresses increased survival to anoxia in neural preconditioned animals, suggesting that HYL-2 functions upstream of the circuit that regulates neural preconditioning.

**Figure 1. hyl-2 is required for neural preconditioning to anoxia:**
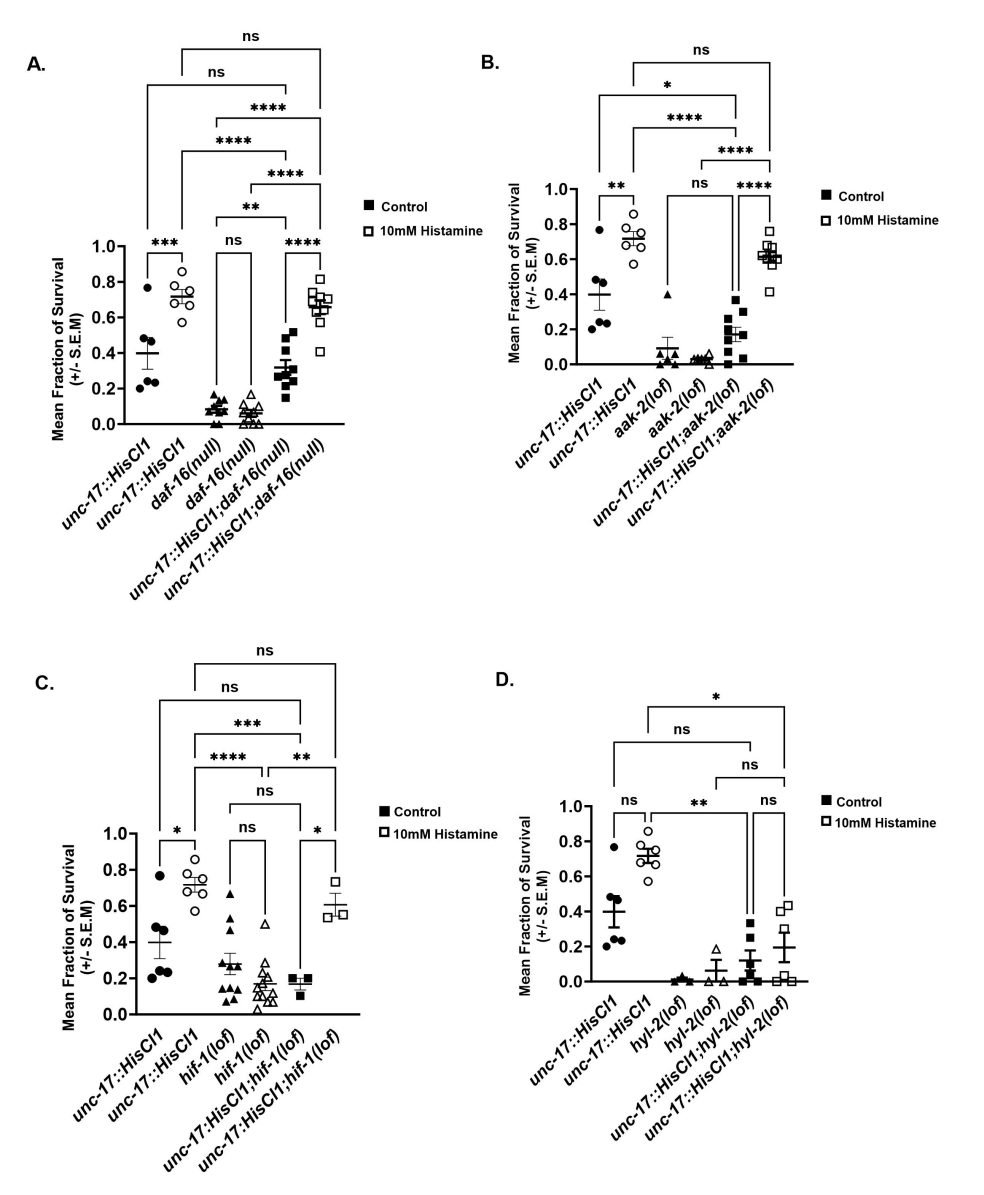
**A.)** Thirty animals carrying the histamine gated chloride channel behind the cholinergic promoter, *unc-17 ,* animals expressing the histamine channel in cholinergic neurons in *daf-16 ( mgDf50 null) background or daf-16 ( mgDf50 null) controls* were selected as early L4 animals to NGM *OP50 E. coli* control plates (black filled shapes) or NGM *OP50 E. coli* experimental plates containing 10 mM histamine plates (non-filled shapes) for 3.5 hours. Animals were allowed to recover on non-histamine plates for 1.5 h and then asphyxiated for 48 hours prior to 48 hours anoxic stress. Mean fraction of survival was scored for animals that developed into adults, regained movement and resumed feeding 24 hours after anoxia. * denotes significance, p value > 0.0001 assessed by ANOVA followed by a Tukey post hoc-test; error bars represent the SEM. Results are shown for 2 independent trials, n=180 animals per condition. **B.)** Wild type animals, animals expressing the histamine channel behind unc-17 promoter, expressing in cholinergic neurons in *aak-2 ( ok524 lf)* background. Experimental design and analysis as in panel A. ANOVA, followed by a Tukey's post-hoc test; error bars represent the SEM. Results are shown for 4 independent trials, n=360 animals per condition. * denotes statistical significance, determined as in panel A, of p = 0.0001 *unc-17p::HisCl1; aak-2 ( ok524 lf)* control versus *unc-17p::HisCl1; aak-2 ( ok524 lf)* 10mM Histamine treatment. **C.)** Loss of *hif-1* does not perturb the cholinergic preconditioning response. Experimental design and analysis as in panel A. p = 0.02 *unc-17p::HisCl1; hif-1 ia4 ) control versus unc-17p::HisCl1; hif-1 ( ia4 lf);* no statistical significance between *hif-1 ( ia4 )* and *unc-17p::HisCl1; hif-1 ( ia4 )* control. **D.)** Loss in *hyl-2* eliminates increased survival to cholinergic preconditioning. Experimental design and analysis as in panel A. Results are shown for 2 independent trials, n=180 animals per condition. No statistical significance between *unc-17p::HisCl1; hyl-2 ( tm2031 ) control versus unc-17p::HisCl1; hyl-2 ( tm2031 )* 10mM Histamine treatment.

## Description

*C. elegans* can be preconditioned to anoxia. Preconditioning can be elicited by chemical, genetic, or environmental conditions. We have previously shown that hyperpolarization of cholinergic neuron activity for 3.5 hours, followed by 1.5 hours of recovery where animals regained locomotor ability, prior to 48 hours of anoxia protects animals and increases survival in L4 stage animals. However, the underlying cellular and molecular mechanisms that confer resistance and increase survival have not been fully established.

We hypothesized that the cholinergic neural preconditioning response is mediated by genes and pathways previously implicated in cellular stress responses. We prioritized candidates based on described roles in *C. elegans* hypoxia and anoxia preconditioning response and generated animals expressing the histamine gated chloride channel, from *Drosophila* , behind the *C. elegans* cholinergic promoter *unc-17* (ch-HisCl1) in the background of a loss of function mutation in one of the stress response genes. The histamine gated chloride channel system has been used previously to selectively and spatially induce silencing of *C. elegans* neurons or neural circuits (Bennett et al., 2021; Pokala et al., 2014) . Using the inducible histamine gated chloride system, we tested *daf-16 ( mgDf50* ), a FOXO transcription factor involved in insulin signaling and mediator of various stress responses; *hif-1 ( ia4 )* the homolog of hypoxia inducible factor gene; *aak-2 ( ok524 ),* the ortholog of AMP activated kinase protein; and *hyl-2 ( tm2031 ),* the ortholog of ceramide synthase gene.

As previously reported, we find that hyperpolarization of cholinergic neuron activity 3.5 hours prior to 48 hours of anoxia, compared to control animals that were not preconditioned resulted in increased survival to anoxia in larval stage 4 (L4) animals (ch-HisCl1 NGM-H- 0.39, ±0.08 versus ch-HisCl1 NGM-H+ 0.71, ±0.04 ANOVA F(5,42) = 44.74 p value < 0.0001, Figure 1A (Bennett et al., 2021) . We tested if a loss in *daf-16* , a FOXO transcription factor which is involved in the insulin-like signaling pathway and is required for DAF-2 insulin receptor mediated survival and adaption of lethal hypoxia at high temperatures, also regulates the cholinergic preconditioning response to anoxia (Mendenhall et al., 2006; Panowski and Dillin, 2009; Scott et al., 2002) . When *daf-16 mgDf50* null animals were placed on either NGM agar plates supplemented with 10 mM histamine (NGM-H+) or control plates lacking histamine (NGM-H-), we find loss of *daf-16* decreases survival to 48 hours of anoxia. However, animals expressing the histamine gated chloride channels in cholinergic neurons *(ch-HisCl1)* in *daf-16* ( *daf-16 ( mgDf50* ) null background did not suppress the increased survival to anoxia in cholinergic preconditioned animals ( *ch-HisCl1; daf-16 ( mgDf50* ) NGM-H- 0.32 ± 0.04 versus *ch-HisCl1; daf-16 ( mgDf50* ) NGM-H+ 0.66± 0.03) Figure 1A . This result suggests that DAF-16 is not required to mediate the cholinergic preconditioning response to anoxia.

In eukaryotes, serine/threonine AMP-activated protein kinase (AMPK) functions as a sensor of energy levels (Witters and Kemp, 1992) . AMPK is a heterotrimeric complex consisting of the α catalytic subunit and regulatory subunits, β and ƴ. *aak-2* encodes the catalytic α subunit of AMPK and regulates longevity and many stress responses in *C. elegans* . (Apfeld et al., 2004; Curtis et al., 2006; Lee et al., 2008; Narbonne and Roy, 2009) . Moreover, specific AMPK subunits are required for environmental induced preconditioning to anoxia in *C. elegans* (LaRue and Padilla, 2011) . Therefore, we hypothesized that environmental and cholinergic neural preconditioning may be co-regulated by AMPK. To determine if AMPK α subunit, *aak-2* was required to mediate cholinergic neural preconditioning to anoxia, we studied animals expressing the histamine gated chloride channel in cholinergic neurons *(ch::HisCl1)* in the *aak-2 ( ok524 )* loss of function mutant background. We find *aak-2 ( ok524 )* loss of function animals appeared normal on NGM-H- control and NGM-H+ plates. Consistent with the findings of LaRue and Padilla, we find loss of *aak-2* decreases survival to 48 hours of anoxia, regardless of whether animals were placed on NGM-H- or NGM-H+ plates ( Figure 1B ). However, ch-HisCl1; *aak-2 ( ok524* ) animals placed on NGM-H+ with loss in cholinergic activity for 3.5 hours prior to 48 hours of anoxia failed to suppress increased survival in cholinergic preconditioned animals compared to controls (ANOVA F _(5,28)_ =34.02, *ch-HisCl1; aak-2 ( ok524* ) NGM-H- 0.17 ± 0.04 versus *ch-HisCl1; aak-2 ( ok524* ) NGM-H+ 0.61± 0.03 Figure 1B . This result suggests that the cellular and molecular mechanisms that regulate environmentally induced preconditioning are distinct from the cellular mechanisms that regulate cholinergic preconditioning to anoxia.

In *C. elegans* survival to hypoxic environments is dependent upon hypoxia-inducible factor (HIF) *hif-1* (Jiang et al., 2001; Shen et al., 2005) . While *hif-1* expression is not essential for survival to anoxia, (Miller and Roth, 2009) we speculated that HIF-1 may still be required for cholinergic preconditioning to anoxia. We find silencing cholinergic neurons in *hif-1* loss of function mutant animals did not increase survival to 48 hours of anoxia. This result is consistent with earlier studies, that *hif-1* is not essential for survival to anoxia. We find that hyperpolarization of cholinergic neurons in ch-HisCl1; *hif-1 ( ia4 )* animals 3.5 hours prior to 48 hours of anoxia did not suppress survival to anoxia (ANOVA F _(5,35)_ =12.4, *ch-HisCl1; hif-1 ( ia4* ) NGM-H- 0.16 ± 0.03 versus *ch-HisCl1; hif-1 ( ia4* ) NGM-H+ 0.60± 0.06, Figure 1C . This result suggests that *hif-1* is dispensable for the cholinergic preconditioning response to anoxia.

Ceramides are the precursors for sphingolipids such as sphingomyelin and glycosphingolipids and are required for membrane structure and mediate cell responses including cell differentiation, apoptosis, and cellular stress (Stiban et al., 2010) . Sphingosine-based ceramides are produced from dihydroceramide in a desaturation step that introduces a trans double bond in sphingoid bases, sphinganine, and serves as the backbone for all sphingolipids (Michel et al., 1997; Mosbech et al., 2013) . Ceramide synthases combine different fatty acyl–coenzyme A (CoA) species to N-acylate sphingoid bases to form dihydroceramide from sphinganine, this is desaturated to form ceramide. Ceramides can be synthesized de novo from palmitate and serine through a series of reactions and is then converted to dihydrosphingosine, this is acylated to form dihydroceramide by ceramide synthases (Michel et al., 1997; Mullen et al., 2012) .

*hyl-2* encodes one of three ceramide synthase genes in *C. elegans* and incorporates fatty acyl side chains lengths (C19 to C23) into ceramides that are processed into sphingomyelin (Menuz et al., 2009; Mosbech et al., 2013) . *hyl-2* mutant animals show increased sensitivity to anoxia and have reduced amounts of ceramides and sphingomyelins with fatty acyl chains of C20 to C22 but have more of ceramides and sphingomyelins species with fatty acyl chains of C24 to C26 (Hannich et al., 2019; Menuz et al., 2009) .

We therefore tested if HYL-2 mediated the cholinergic preconditioning response to anoxia. To determine if *hyl-2* was required for cholinergic preconditioning, we studied animals expressing the histamine gated chloride channel in cholinergic neurons *(ch::HisCl1)* in the *hyl-2* ( *hyl-2 ( tm2031* ) loss of function mutant background. Consistent with what was reported by Menuz et al., loss in *hyl-2* decreased survival to 48 hours of anoxia. We find the increased survival of cholinergic preconditioned animals is suppressed in *hyl-2* loss of function mutant animals (ANOVA F _(1.4,7)_ =13.74, *ch-HisCl1; hyl-2 ( tm2031* ) NGM-H- 0.11 ± 0.05 versus *ch-HisCl1; hyl-2 ( tm2031* ) NGM-H+ 0.19 ± 0.08, Figure 1D . This result suggests that *hyl-2* functions upstream of the genetic or neural circuit that confers increased survival to anoxia in neuronally preconditioned animals.

Given HYL-2 established roles in mediating various cell activities, these findings also bolster support that HYL-2 mediates several stress responses, including anoxic stress.

## Methods

**Strains**

**Table:** 

**Strain**	**Gene (allele)/genotype**	**Oligos**
N2		
GR1307	*daf-16 ( mgDf50 ) I *	
ZG31	*hif-1 ( ia4 ) V *	
RB754	*aak-2 ( ok524 )* X	
HLB1	*hyl-2 ( tm2031 )X *	Backcrossed twice IntRev: cactgctctactgataacac Int Fwd: ccgttaacagaagcatgatg ExtRev: aggcagaactgccgtcgttc ExtFwd: atacgcattggtgacaggta
HLB2	*hlwEx1 [pJP673(Punc-17::HisCl1; myo-2p::mCherry)] *	
HLB3	*hlwEx1 [pJP673(Punc-17::HisCl1; myo-2p::mCherry)]* ; *aak-2 ( ok524 )X *	Fwd Primer: cccaatctgccaaatactgac Rev Primer (outer): cacgaccatacatcaacttcg Rev Primer (inner): cattgttctgctcatcgagc
HLB4	*hlwEx1 [pJP673(Punc-17::HisCl1; myo-2p::mCherry)]; daf-16 ( mgDf50 ) I *	Fwd Primer: ctctctctgtttctccccgc Rev Primer (outer): acggacactgttcaactcgt Rev Primer (inner): gcgagagtagcgatgttgga
HLB5	*hlwEx1 [pJP673(Punc-17::HisCl1; myo-2p::mCherry)]* ; *hif-1 ( ia4 ) V *	Fwd Primer: gaatgccgcatgttccgatc Rev Primer (outer): cggagcagcaatacaagatg Rev Primer (inner): atggtgtcttcagtccatacc
HLB6	*hlwEx1 [pJP673(Punc-17::HisCl1; myo-2p::mCherry)]; hyl-2 ( tm2031 ) X *	IntRev: cactgctctactgataacac Int Fwd: ccgttaacagaagcatgatg ExtRev: aggcagaactgccgtcgttc ExtFwd: atacgcattggtgacaggta

***C. elegans* husbandry and media preparation**

Strains were reared on NGM plates seeded with OP50 *E. coli* under standard conditions. NGM-HA plates were prepared as described in (Pokala et al., 2014) . Synchronous populations were generated by bleaching gravid adults and two days later L4 stage animals were collected and assayed for survival to anoxia (Stiernagle, 2006) . Some strains were obtained directly from CGC, others were obtained from the National BioResource Project in Tokyo Japan. These strains were backcrossed at least twice in the Kalb lab before generating transgenic strains; no additional backcrossing was done in the described experiments. Please note, strain HLB2 was previously listed as RK206 and published in Bennett et al., Genes, Brain, and Behavior in 2021. This strain is now listed as HLB2 .

**Exposure to histamine to illicit neural preconditioning paradigm**

Animals were selected as early L4 stage animals, as characterized by vulval indentation, and placed to either NGM plates containing 10mM of histamine or control plates lacking histamine for 3.5 hours. Animals transferred to NGM plates lacking histamine for 1.5 hours prior to anoxia exposure. Experimental design and procedure as previously described in Bennett et al., 2021. Animals expressing the histamine chloride channel behind a cholinergic specific promoter *unc-17* , (ch-HisCl1) when exposed to 10mM histamine became paralyzed in 2 to 5mins.

**Anoxia exposure and assessment of survival**

All experiments were done with L4 stage animals. For anoxic insult, 30 mid L4 stage animals per genotype were selected and exposed to anoxia via Bio-Bag ^TM^ (Type A anaerobic environmental system, Becton-Dickinson). Anaerobic anoxia environments were induced and confirmed by a resazurin indicator, as previously described by (Doshi et al., 2019) . Animals were exposed to 48 hours of anoxia at 20°C, then re-exposed to oxygen at 20°C for 24 hours. After 24 hours animals were scored for survival. Survival was determined for animals that developed into adults, regained movement and/or resumed feeding after 24 hours after re-oxygenation as described in Flibotte et al., 2014.

**Statistical analysis**

Statistical analysis was performed using GraphPad Prism version 9 (GraphPad Software, La Jolla, CA). Experiments are done in triplicate with 30 animals per genotype or condition and independent trials are done on different days, therefore survival results were pooled from data collected from at least two independent trials. We report the standard deviation and standard error of mean for all experiments. The mean survival was analyzed by one-way ANOVA, followed by a Tukey's multiple comparisons post hoc test and significance was determined as p< 0.05.
